# Impact of microwave- and far-infrared-assisted acid modification on the structure and functional properties of zein-corn dough

**DOI:** 10.1016/j.fochx.2026.104159

**Published:** 2026-07-02

**Authors:** Yanjia Liu, Zhuang Liu, Bin Song, Qianqian Li, Yu Wang, Yuhan Zhou, Chengbin Zhao, Hao Zhang, Yuzhu Wu, Xiuying Xu, Jingsheng Liu

**Affiliations:** a College of Food Science and Engineering, Jilin Agricultural University, Changchun, Jilin 130118, China; b National Engineering Research Center of Wheat and Corn Further Processing, Changchun, Jilin 130118, China

**Keywords:** Far-infrared modification, Gluten-free dough, Microwave modification, Protein-starch interaction, Zein

## Abstract

Gluten-free corn dough suffers from a significant imbalance between mechanical strength and ductility, a fundamental limitation stemming from the lack of a continuous gluten network. In this study, zein was modified through synergistic microwave-assisted acid and far–infrared-assisted acid treatments to produce high-quality gluten-free corn dough. Compared to the control, the far-infrared-assisted acid modified dough demonstrated a 144% increase in β-sheet content, a 109 - fold rise in storage modulus, a 90.70% enhancement in hardness, and a 19.95% elevation in thermal denaturation peak temperature. These enhancements were attributed to the synergistic effects of disulfide cross-linking and non-covalent interactions (hydrogen bonds and hydrophobic interactions), achieving an optimal balance between strength and ductility with a maximum tensile elongation of 49.70 mm. Furthermore, the microwave-assisted acid modified sample exhibited favorable tensile strength that satisfies the practical requirements for baking. This research presents a straightforward, feasible, and effective strategy for developing high-performance gluten-free cereal products.

## Highlights

• Novel green microwave-acid & far-infrared-acid synergistic methods tailor zein for gluten-free corn dough.
• Far-infrared-acid treatment lifted β-sheet by 144% and storage modulus 109-fold vs untreated control dough.
• Covalent & non-covalent synergies from far-infrared-acid treatment delivered balanced dough strength and ductility.
• Microwave-acid modified dough owns excellent tensile strength, showing promising application value in baking production.
• We systematically clarified distinct regulatory mechanisms of the two thermal treatments on zein-based gluten-free dough.

## Graphical abstract

**Figure:**
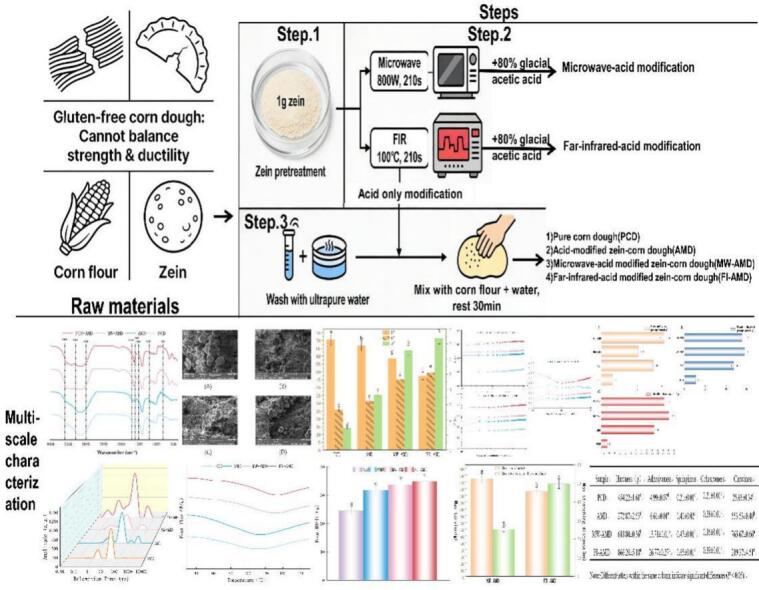

## 1 Introduction

The increasing prevalence of celiac disease (CD), non-celiac gluten sensitivity (NCGS), and wheat allergy (WA) has spurred the rapid growth of the global gluten-free food market. This market is anticipated to expand at a compound annual growth rate (CAGR) of 7.2% from 2024 to 2030 (Piec & Jancza-Smuga, 2022; Singla et al., 2024). Wheat dough, a staple in cereal-based foods, exhibits unique viscoelasticity and ductility. These characteristics arise from a continuous three-dimensional network formed by the cross-linking of glutenin and gliadin (Wang et al., 2022; Zang et al., 2022). In contrast, gluten-free dough lacks this natural protein matrix, resulting in significant quality deficiencies, including a loose and discontinuous structure, poor water retention, inadequate thermal stability, and unbalanced mechanical performance (Capriles et al., 2023). Such limitations hinder the processability and consumer acceptance of gluten-free cereal products, making their resolution a central focus of research in gluten-free food processing.

Current strategies for enhancing the quality of gluten-free dough primarily involve the incorporation of hydrophilic colloids, emulsifiers, dietary fiber, and alternative plant proteins (Mir, Riar, & Singh, 2021; Skendi et al., 2021; Tahmouzi et al., 2025). Among these, the addition of zein has emerged as the most promising method. Zein, the predominant storage protein in corn, is a GRAS-certified (Generally Recognized as Safe), widely available, and safe gluten-free alternative protein. Under thermal, solvent, or mechanical shear treatments, zein can fold and aggregate through hydrophobic interactions, resulting in the formation of nanofibrous or film-like structures. These structures can replicate the network-forming ability of gluten, encapsulate starch granules, and enhance the viscoelastic properties of gluten-free dough (Akshorayeva et al., 2023; Sadat et al., 2023). To further enhance the network-forming capacity and compatibility of zein in dough, various modification techniques have been developed. Physical modification methods, including high-pressure treatment, ultrasonic treatment, microwave treatment, and far-infrared (FIR) radiation, have garnered significant attention due to their advantages of environmentally friendly processing, high efficiency, and the absence of chemical reagent residues, particularly when compared to chemical and enzymatic modifications (Hu et al., 2024; Li et al., 2021; Zheng et al., 2024) Existing studies have demonstrated that microwave treatment can modify the secondary and tertiary structures of zein, expose active functional groups, and enhance its intermolecular cross-linking capacity (Wang et al., 2023). However, research on the structural modification of zein through far-infrared (FIR) treatment remains limited. Its application in gluten-free dough systems has also been infrequently reported (Hu et al., 2024). Current investigations primarily concentrate on the isolated effects of microwave modification on the functional properties of zein. There is a notable absence of systematic comparisons regarding the differential regulatory mechanisms of these two thermal fields on zein conformation and intermolecular interactions. Most studies focus solely on the direct effects of modified zein on dough quality. They do not clarify the comprehensive regulatory mechanism, which involves protein conformation rearrangement, enhanced protein-starch interfacial interactions, microstructural remodeling, and improved macroscopic processing performance.

In this study, zein was modified through microwave-acid and FIR-acid synergistic treatments, and subsequently blended with corn flour to produce gluten-free corn dough. For dough samples containing differently modified zein, several indicators were systematically assessed. These indicators included the secondary structure, sulfhydryl-disulfide bond content, and surface hydrophobicity of zein, as well as color difference, microstructure, thermal stability, water distribution, dynamic rheological properties, textural characteristics, and tensile properties of the dough. We conducted a Pearson correlation analysis to elucidate the quantitative relationship between zein structural changes and the evolution of dough quality. This analysis aimed to uncover the regulatory mechanisms by which various modification treatments affect the functional properties of gluten-free dough. This study's novelty resides in elucidating the distinct regulatory mechanisms underlying the synergistic modification of zein-based gluten-free dough by microwave and far-infrared (FIR) treatments. Microwave treatment primarily enhanced dough tensile strength by promoting high-density disulfide covalent cross-linking. In contrast, FIR treatment established an optimal balance between dough strength and ductility through the synergistic enhancement of both covalent disulfide cross-linking and non-covalent interactions, including hydrogen bonds and hydrophobic interactions. This study addresses the existing research gap regarding the differential effects of various thermal physical fields on zein conformation. It systematically clarifies the complete regulatory pathway from protein conformational rearrangement to macroscopic dough processing performance. Furthermore, it establishes a quantitative correlation between zein conformation and dough processing properties, thereby providing an environmentally friendly and efficient theoretical foundation and technical support for the development of high-quality gluten-free cereal staple foods.

## 2 Materials and instruments

### 2.1 Materials and reagents

Corn flour was prepared by cleaning, grinding and sieving Jinongyu 918 corn kernels (purchased from a local seed market in Changchun, China) through a 200-mesh sieve, with a moisture content of 10.21% (wet basis), crude protein content of 8.98% and crude starch content of 71.71% (both on a dry basis). Zein (purity ≥98%; Product No. 294946) was obtained from J&K Scientific Ltd., Beijing, China. Glacial acetic acid, hydrochloric acid, sodium hydroxide, 5,5′-dithiobis-(2-nitrobenzoic acid) (DTNB), and other analytical-grade chemical reagents were all supplied by Sinopharm Chemical Reagent Co., Ltd., Shanghai, China.

### 2.2 Preparation of modified zein and gluten-free corn dough

#### 2.2.1 Preparation of modified zein

See Fig. 1.

**Fig. 1:**
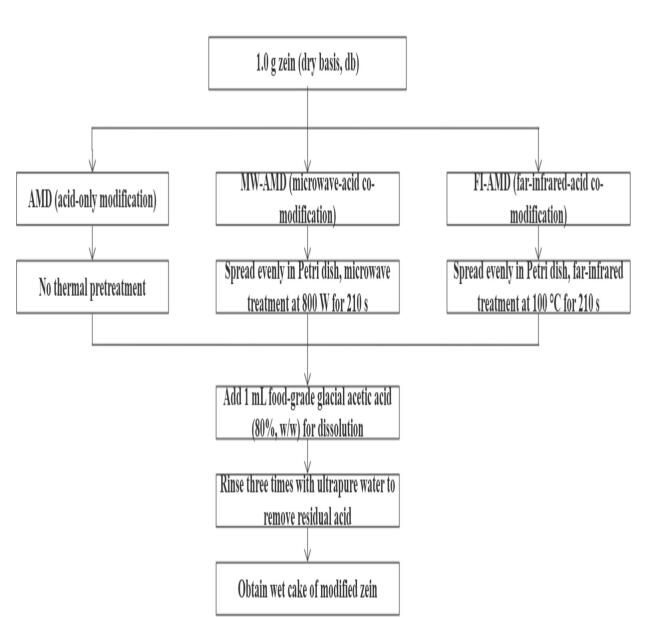
Flowchart for the preparation of three kinds of modified zein.

#### 2.2.2 Preparation of gluten-free corn dough

**Fig. 2:**
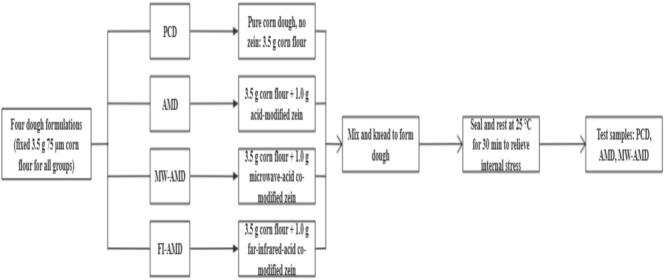
Flowchart for the preparation of four groups of gluten-free corn dough.

All dough samples adopted the unified total water addition of 3.0 mL ultrapure water. For groups supplemented with modified zein wet cake, the moisture contained in the filter cake was deducted from the supplementary water to ensure consistent moisture content across all treatments (See Fig. 2).

### 2.3 Protein secondary structure analysis by Fourier transform infrared spectroscopy (FTIR)

The secondary structure of zein in the dough system was determined using an FTIR spectrometer (Tensor II, Bruker Optics GmbH, Karlsruhe, Germany). The dough sample was freeze-dried, ground, and mixed with KBr at a mass ratio of 1:100, then pressed into a KBr pellet. The pellet was scanned over a wavenumber range of 4000–500 cm^−1^, with a resolution of 4 cm^−1^ and 64 scans. Infrared spectra were processed and plotted using Origin 2021 (OriginLab Corporation, Northampton, MA, USA). The amide I band (1700–1600 cm^−1^) was deconvoluted and fitted using PeakFit 4.12 software (SeaSolve Software Inc., Framingham, MA, USA), and the relative contents of α-helix, β-sheet, β-turn, and random coil were calculated based on peak area (Lan et al., 2026).

### 2.4 Determination of free sulfhydryl and disulfide bond content

The free sulfhydryl (SH) and disulfide bond (SS) contents of dough samples were determined as described by Kang et al. (Kang et al., 2024). Freeze-dried dough powder was dissolved and made up to 10 mL with guanidine hydrochloride-containing buffer to prepare the sample solution. For free SH content measurement, the sample solution was mixed with urea-guanidine hydrochloride solution and Ellman's reagent. The absorbance was measured at 412 nm using a UV–visible spectrophotometer (UV-1800, Shimadzu Corporation, Kyoto, Japan). The sulfhydryl content was calculated using the following formula:

$$C_{\mathrm{SH}}=\frac{73.53A_{412}D}{C_{S}}$$

In the equation: *C*_*SH*_ represents the thiol content, μmol/g; *A*_*412*_ represents the absorbance at 412 nm; *C*_*s*_ represents the mass concentration of the sample, mg/mL; *D* represents the dilution factor.

For total SH content, the sample solution was reduced with β-mercaptoethanol, precipitated with trichloroacetic acid (TCA), washed, and redissolved in 8 mol/L urea solution, then reacted with Ellman's reagent, followed by absorbance measurement at 412 nm. The disulfide bond content was calculated using the following formula:

$$\;C_{\mathrm{SS}}=\frac{C_{\mathrm{SH}\left(\text{Total}\right)-}C_{\mathrm{SH}\left(\text{Free}\right)}}{2}$$

In the equation: *C*_*ss*_ is the disulfide bond content, μmol/g; *C*_*SH(Total)*_ is the total sulfhydryl content, μmol/g; *C*_*SH(Free)*_ is the free sulfhydryl content, μmol/g.

### 2.5 Determination of surface hydrophobicity

The surface hydrophobicity of proteins in dough samples was determined using a modified bromophenol blue (BPB) binding assay (Zhu et al., 2025). Dough samples were suspended in 0.1 M phosphate buffer (pH 7.0), mixed with BPB solution, vortexed for 10 min at room temperature, and centrifuged at 6000 ×*g* for 10 min. The absorbance of the supernatant was measured at 595 nm with a microplate reader (Feyond-A500, Hangzhou Aosheng Instrument Co., Ltd., Hangzhou, China). A matrix blank control was set to eliminate non-protein interference, and surface hydrophobicity was expressed as the amount of bound BPB.

$$H_{0}\left(\mathrm{\mu g}\right)=\frac{40\times \left(A_{\text{Blank}}-A_{\text{Sample}}\right)}{A_{\text{Blank}}}$$

In the equation: *H*_*0*_ represents surface hydrophobicity (μg); *A*_*Blank*_ is the absorbance of the blank control at 595 nm; *A*_*Sample*_ is the absorbance of the sample at 595 nm; 40 is a constant coefficient.

### 2.6 Microstructure observation by scanning Electron microscopy (SEM)

Dough specimens were fixed onto a sample stage with a diameter of 10 mm, sputter-coated with gold for 3 s, and observed under a scanning electron microscope (SU8010, Hitachi High-Technologies Corporation, Tokyo, Japan) at an accelerating voltage of 5 kV (de Oliveira et al., 2020).

### 2.7 Color difference analysis

The color of the dough sample was measured using a colorimeter (CR-400, Konica Minolta Inc., Tokyo, Japan) which was pre-calibrated with a standard white plate before each measurement. The recorded CIE Lab color parameters included *L*^*⁎*^ (lightness), *a*^*⁎*^ (red-green value), and *b*^*⁎*^ (yellow-blue value) (Giannoutsos et al., 2023). The total color difference (ΔE) between each modified dough sample and the control (pure corn dough) was calculated using the following formula:

$$\mathrm{\Delta E}=\sqrt{{\left(L^{*}-{L_{0}}^{*}\right)}^{2}+{\left(a^{*}-{a_{0}}^{*}\right)}^{2}+{\left(b^{*}-{b_{0}}^{*}\right)}^{2}}$$

where *L*_*0*_^*⁎*^, *a*_*0*_^*⁎*^, and *b*_*0*_^*⁎*^ are the color parameters of the control sample.

### 2.8 Analysis of water distribution by low-field nuclear magnetic resonance (LF-NMR)

Water distribution and mobility of dough were measured using an LF-NMR analyzer (MesoMR23-060H-I, Niumag Electronic Technology Co., Ltd., Shanghai, China). A 4 g dough sample was placed in an NMR tube, and the Carr-Purcell-Meiboom-Gill (CPMG) sequence was used. The test parameters were set as follows: resonance frequency 23.4 MHz, 90^°^ pulse width 13.2 μs, 180^°^ pulse width 25.4 μs, delay time 3000 ms, echo time 0.2 ms, number of echoes 12,000, and 16 scans. The transverse relaxation time (*T*_*2*_) and the corresponding signal amplitude (*A*_*2*_) were determined to characterize water mobility in the sample.

### 2.9 Thermal stability analysis by differential scanning calorimetry (DSC)

The thermal stability of the dough was determined using a differential scanning calorimeter (DSC 25, TA Instruments, New Castle, DE, USA). 3.00 ± 0.02 mg of dough sample was accurately weighed into an aluminum crucible, sealed, and scanned from 30 °C to 180 °C at a heating rate of 10 °C/min, with nitrogen as the protective gas at a flow rate of 50 mL/min. An empty sealed aluminum crucible was used as the reference. The thermal denaturation peak temperature (*T*_*p*_) and enthalpy (*ΔH*) values were recorded from the thermogram (Mu et al., 2025).

### 2.10 Dynamic rheological properties measurement

The dynamic rheological properties of the dough were determined using a rotary rheometer (MCR 302, Anton Paar, Graz, Austria) with a parallel plate fixture (25 mm diameter, 1 mm gap). The dough sample was placed between the fixtures, equilibrated for 5 min to eliminate residual stress, and tested at 25 °C.

1) Frequency sweep test: The strain was fixed at 0.5% (within the linear viscoelastic region), and the angular frequency range was 0.1–100 rad/s. The storage modulus (*G'*) and loss modulus (*G"*) of the sample were recorded.

2) Strain sweep test: The angular frequency was fixed at 10 rad/s, and the strain range was 0.01–100% to determine the linear viscoelastic region of the sample (Zhou et al., 2017).

### 2.11 Texture profile analysis (TPA)

The texture characteristics of the dough were measured using a texture analyzer (TA-XT Plus, Stable Micro Systems, Surrey, UK) equipped with a P/36R cylindrical probe. Freshly prepared dough samples were cut into standard-sized pieces (2 mm × 10 mm × 30 mm). The test parameters were set as follows: pre-test speed 1.00 mm/s, test speed 5.00 mm/s, post-test speed 5.00 mm/s, compression ratio 50%, trigger force 5 g, and two compression cycles. The hardness, adhesiveness, springiness, cohesiveness, and chewiness of the dough were recorded (van der Graaf et al., 2026).

### 2.12 Tensile properties measurement

The tensile properties of the dough were measured using the texture analyzer (TA-XT Plus, Stable Micro Systems, Surrey, UK) equipped with a Kieffer dough and gluten extensibility rig. The dough sample was rolled into a 2 mm thick sheet, cut into a standard tensile mold, and equilibrated at 25 °C for 10 min before testing. The test parameters were set as follows: initial grip distance, 20 mm; tensile speed, 50 mm/min; trigger force 5 g. The maximum stretching distance and tensile resistance of the dough were recorded (van der Graaf et al., 2026).

### 2.13 Pearson correlation analysis

Pearson correlation analysis was conducted using SPSS 26.0 software (IBM, Armonk, NY, USA) to investigate the linear correlation between two categories of parameters: zein structural characteristics and gluten-free dough quality properties. The zein structural characteristics encompassed secondary structure components, free sulfhydryl content, disulfide bond content, and surface hydrophobicity. The gluten-free dough quality properties included color difference, textural parameters, dynamic rheological properties, tensile properties, thermal stability, and water distribution. Prior to the correlation analysis, the normality of all continuous variables was assessed using the Shapiro-Wilk test, confirming that all variables satisfied the normal distribution criterion (*P* = 0.062–0.918, all >0.05). The Pearson correlation coefficient (*r*) was calculated to quantify the strength and direction of the linear relationship between variables, and the significance of the correlation was tested at two levels: ^⁎^*P* < 0.05 (significant correlation) and ^⁎⁎^*P* < 0.01 (extremely significant correlation). All analyses were performed based on 3 independent biological replicates for each index.

### 2.14 Statistical analysis

All experiments were performed in triplicate, and the data were expressed as mean ± standard deviation (SD). The normality and homogeneity of variance of all data were verified by Shapiro-Wilk test and Levene's test, respectively. Data were subjected to one-way ANOVA using SPSS 26.0, and mean comparisons were conducted using Duncan's test (*P* < 0.05).

## 3 Results and discussion

### 3.1 Effects of different modifications on zein secondary structure in dough

**Fig. 3:**
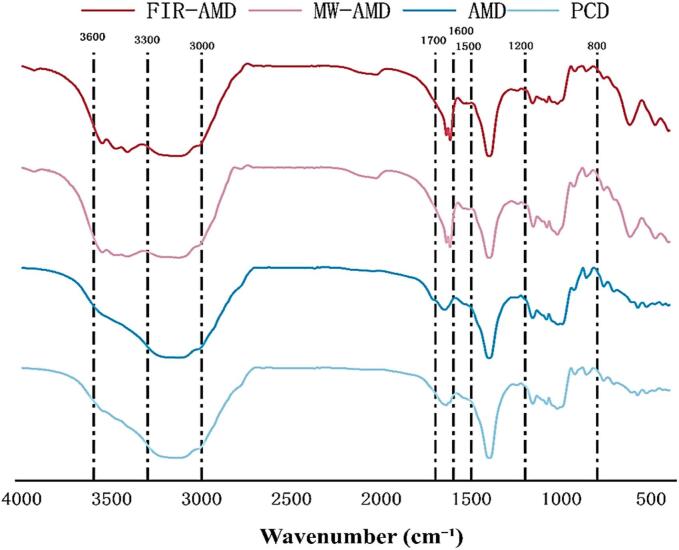
FTIR spectra of gluten-free zein-corn dough.

FTIR spectra (Fig. 3) revealed the changes in molecular interactions and functional groups of dough samples. In the 3600–3000 cm^−1^ region (corresponding to O-H/N-H stretching vibration), MW-AMD and FI-AMD showed stronger and blue-shifted absorption peaks compared with PCD and AMD, indicating enhanced intermolecular hydrogen bonding (Sun et al., 2023). Quantitative analysis based on the peak area of O-H/N-H stretching vibration showed that the relative hydrogen bond content of FI-AMD and MW-AMD increased by 76.3% and 58.7%, respectively, compared with the PCD control. This finding provided direct quantitative evidence that thermal-acid synergistic modification strengthened non-covalent interactions. The amide I band (1700–1600 cm^−1^) of modified samples showed an absorption peak at approximately 1630 cm^−1^, confirming the formation of stable β-sheets. Such structures are crucial to offset the lack of gluten in dough (Mir, Sablania, et al., 2021). In the 1200–900 cm^−1^ region (C—O stretching of polysaccharides), modified samples, especially FI-AMD, displayed stronger absorption peaks. This observation suggested reinforced molecular interactions between zein and starch.

**Fig. 4:**
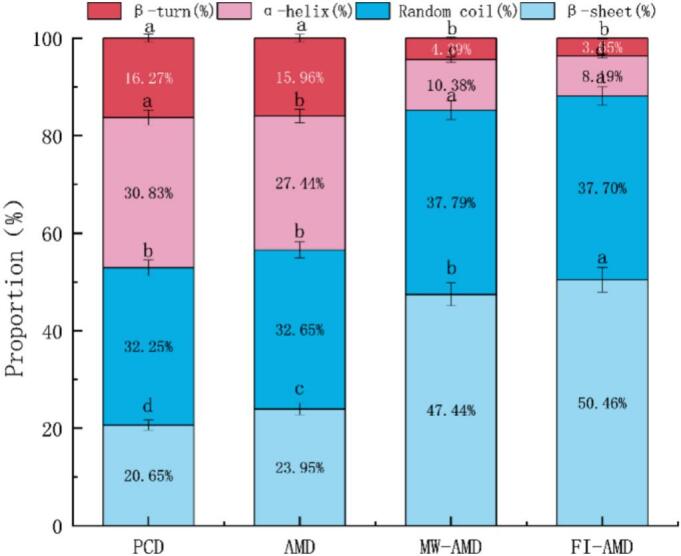
Secondary structure composition of gluten-free zein-corn dough under different treatments.

FTIR analysis revealed that thermal-acid synergistic modification significantly altered the secondary structure of zein in gluten-free dough (Fig. 4). Compared with PCD, MW-AMD and FI-AMD remarkably reduced the contents of α-helix and β-turn. The α-helix content decreased from 30.83 to 8.19, while the β-turn content declined from 16.27 to 3.65%. On the contrary, the β-sheet content increased obviously from 20.65 to 47.44 and 50.46% for MW-AMD and FI-AMD, respectively. FI-AMD presented the highest β-sheet proportion, indicating that far-infrared heating contributed to the formation of more ordered protein aggregates.

The increase in β-sheet content was mainly attributed to thermal-acid synergistic modification. Such treatment disrupted the compact α-helix structure of native zein, promoted the unfolding of protein molecular chains, and further facilitated the formation of intermolecular β-sheet aggregates via hydrogen bonding. This structural rearrangement provides the molecular basis for establishing a stable and continuous protein network in gluten-free dough, and the enriched β-sheet structure is critical to improving network rigidity and processing properties of dough. This observation was in good agreement with the findings reported by Zhang et al. (Zhang et al., 2022), who demonstrated that modified globulin could enhance the structural integrity of the zein network in gluten-free systems.

### 3.2 Effects of different modifications on chemical forces in dough system

**Fig. 5:**
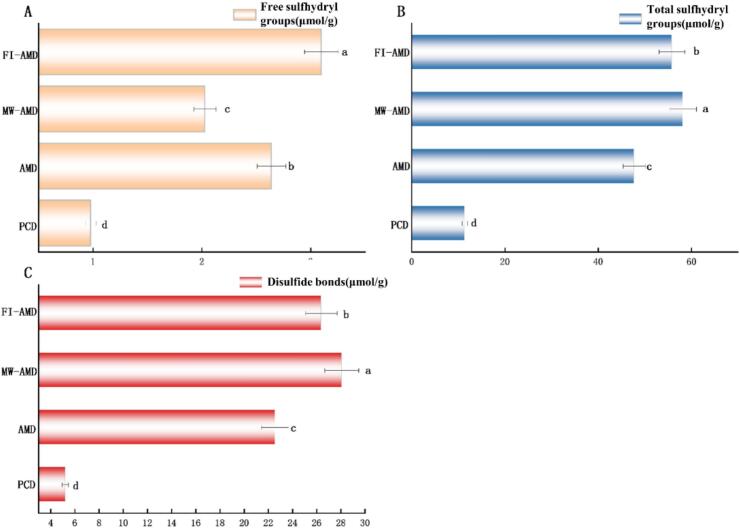
Free sulfhydryl, total sulfhydryl and disulfide bond contents of gluten-free corn dough samples.

Sulfhydryl groups and disulfide bonds are critical covalent forces that maintain the spatial conformation of zein and the three-dimensional network of corn dough (Hu et al., 2026). The contents of free SH, total SH and SS in all samples are presented in Fig. 5. Compared to the PCD control, all zein modification treatments significantly increased the levels of free SH, total SH, and SS in corn dough (*P* < 0.05). Specifically, the free SH content in all modified groups was 2.07**–**3.16 times higher than that of PCD. This increase is attributed to the destruction of hydrogen bonds and hydrophobic interactions of zein due to acid modification and thermal-acid synergistic treatment, which promoted the unfolding of protein molecular chains and exposed free SH groups that were originally embedded in the hydrophobic core of zein. The highest free SH content was observed in FI-AMD (3.10 μmol/g), indicating that the mild and uniform thermal effect of far-infrared heating maximized the unfolding of zein molecules and exposed a greater number of active sulfhydryl groups without inducing excessive protein aggregation.

Microwave induces rapid volumetric heating. This effect causes instantaneous local overheating and rapid oxidation of sulfhydryl groups. By contrast, far infrared delivers uniform and mild radiative heating. Such heating penetrates the protein matrix evenly (Oduro et al., 2021). Far infrared treatment exposes sulfhydryl groups. Meanwhile, this approach preserves the reactivity of hydrophobic amino acid residues. These changes support simultaneous enhancement of covalent and non-covalent interactions (Federici et al., 2021). Total sulfhydryl content reflects all reactive sulfhydryl groups in the system, including those involved in disulfide bonds. Significant increases in modified groups further verify that modification disrupts the compact spherical structure of zein. Such treatments release bound sulfhydryl groups and improve overall protein reactivity.

For SS bonds, modified samples exhibited 3.33–5.39 times higher content than PCD. This trend agreed well with the variation of storage modulus (G′) in rheological tests (Section 3.7). It proves that enhanced disulfide cross linking mainly improves the elastic network and rheological properties of modified corn dough (Giannoutsos et al., 2023). MW-AMD and FI-AMD had higher SS content than AMD. Microwave and far infrared heating further oxidized exposed free SH groups. This reaction forms intermolecular SS bonds. Therefore, a denser and more continuous protein cross linking network is constructed. FI-AMD showed slightly lower SS content than MW-AMD. However, its G′ value was the highest among all samples (Section 3.7). This result suggests two key factors for dough network reinforcement. One is disulfide-mediated covalent cross linking. The other is non covalent interactions strengthened by far infrared treatment, including hydrogen bonds and hydrophobic interactions (Zhou et al., 2014).

**Fig. 6:**
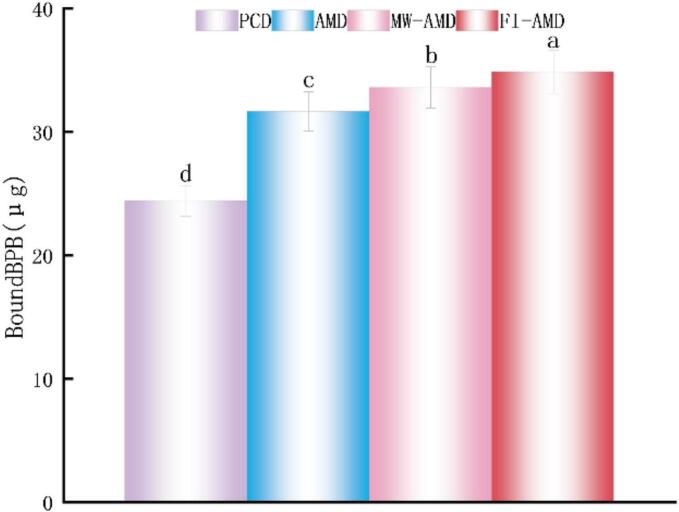
Surface hydrophobicity of gluten-free corn dough samples.

The surface hydrophobicity of proteins in dough is shown in Fig. 6. All zein modification treatments significantly increased the surface hydrophobicity of the dough system compared with PCD (*P* < 0.05). Thermal-acid synergistic treatments achieved a further significant increase in surface hydrophobicity compared with single acid modification (*P* < 0.05). The FI-AMD group has the highest bound BPB value among all samples (33.26 μg/g). Bound BPB value directly quantifies surface hydrophobicity. Far infrared-acid synergistic treatment maximally unfolds zein molecules. More hydrophobic amino acid residues are therefore exposed as a result. FI-AMD showed significantly higher SS content than AMD. Even so, its surface hydrophobicity remained the highest. This result indicates that far-infrared heating dominates zein unfolding. Its effect outweighs the embedding of hydrophobic residues caused by cross-linking (Li et al., 2024). According to FTIR results, increased surface hydrophobicity provides more active sites for intermolecular hydrophobic interactions. Meanwhile, hydrogen bonding was enhanced by 76.3% compared with PCD. This stronger hydrogen bonding further stabilizes the protein network. These changes explain why FI-AMD achieved the highest storage modulus (G'), despite slightly lower SS content than MW-AMD. Higher surface hydrophobicity of modified zein offers active sites for intermolecular hydrophobic interactions. It also exposes free SH groups originally buried in native structures. This exposure accelerates intermolecular SS bond formation. Together, these effects build a compact and continuous protein-starch network.

### 3.3 Effects of different modifications on microstructure of gluten-free dough

**Fig. 7:**
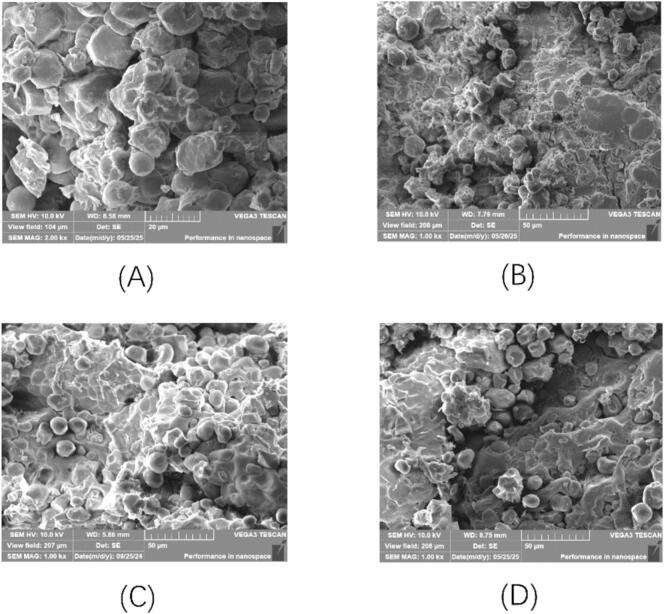
SEM micrographs of gluten-free corn dough with different treatments. Note: (A) Pure corn dough (PCD); (B) Acid-modified zein corn dough (AMD); (C) Microwave-acid modified zein corn dough (MW-AMD); (D) Far-infrared-acid modified zein corn dough (FI-AMD).

The SEM micrographs of gluten-free dough are presented in Fig. 7. PCD displayed intact polygonal starch granules with distinct boundaries. Large voids existed among these granules. This structure indicated weak interfacial interactions between native zein and starch. It thus formed a loose and discontinuous network (Lin et al., 2024) (Fig. 7-A). After AMD treatment, starch granules were partially broken and refined. Their edges became blurred, and internal pores decreased. A more homogeneous but still discontinuous matrix was formed (Fig. 7-B). MW-AMD showed the most compact and continuous microstructure. Its protein dispersion was improved. Strong interfacial bonding formed between zein and starch granules (Fig. 7-C). FI-AMD generated a dense and continuous protein network surrounding starch granules. Minor surface roughness was observed. This phenomenon stemmed from synergistic covalent and non-covalent interactions. Far-infrared heating induced disulfide cross-linking, hydrogen bonds and hydrophobic interactions. These forces strengthened interfacial adhesion between zein and starch mainly via hydrophobic interactions and hydrogen bonds. Accordingly, a more stable composite network was constructed (Fig. 7-D). Microstructural observations were highly consistent with dough rheological and textural properties. These findings confirmed that thermal-acid modification of zein effectively optimizes the microstructure of gluten-free dough.

### 3.4 Effects of different modifications on color parameters of dough

**Fig. 8:**
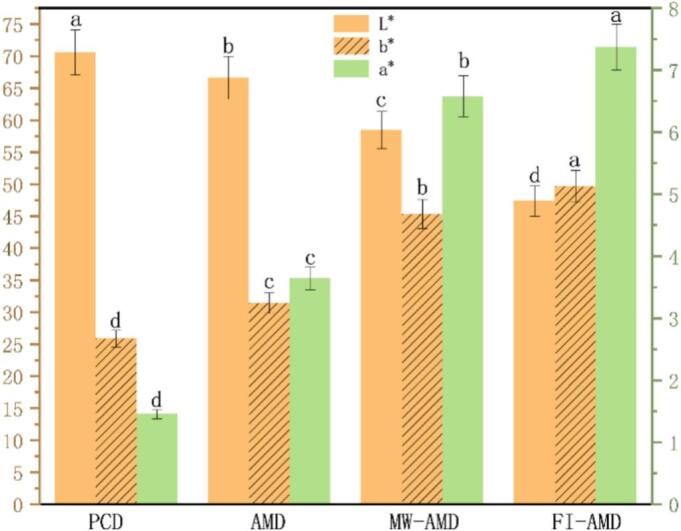
Color parameters (L*, a*, b*) of gluten-free corn dough with different treatments.

The color parameters (L^⁎^, a^⁎^, b^⁎^) of gluten-free dough are presented in Fig. 8. PCD had the highest lightness (L^⁎^). It also showed the lowest redness (a^⁎^) and yellowness (b^⁎^). These values reflect the natural color of native corn starch and zein. Acid modified samples (AMD) showed a partial decline in L^⁎^. Meanwhile, their a^⁎^ and b^⁎^ values increased. This change arose from acid triggered protein unfolding and exposed reducing sugars. These factors initiated non enzymatic browning reactions. Compared with AMD, MW-AMD further reduced lightness. Its a^⁎^ and b^⁎^ values increased significantly. Microwave irradiation exerts synergistic thermal and non thermal effects. These effects accelerate Maillard reactions and promote color formation (Elkatry et al., 2024). FI-AMD exhibited the lowest L* among all groups. It also had the highest a* and b^⁎^ values. Uniform far infrared heating strengthens interactions between modified zein and starch. This process induces more intense non enzymatic browning (Dahal et al., 2020). As shown in Table 1, consistent with the above variations in L*, a* and b*, the total ΔE values of MW-AMD and FI-AMD both exceeded 10, demonstrating extremely remarkable color differences compared with their respective reference samples; notably, FI-AMD exhibited substantially larger overall color deviation than MW-AMD.

**Table 1:** Effect of different thermal modification treatments on total color difference (ΔE) of gluten-free corn dough.

Sample	ΔE_1_	ΔE_2_
MW-AMD	23.48	16.39
FI-AMD	33.75	26.77

Note: Different lowercase letters in the same column represent significant differences at *P* < 0.05; ΔE₁ and ΔE₂ were calculated using JNY group (Control 1) and C group (Control 2) with identical particle sizes as the respective references for MW-AMD and FI-AMD.

### 3.5 Effects of different modifications on water distribution in dough

**Fig. 9:**
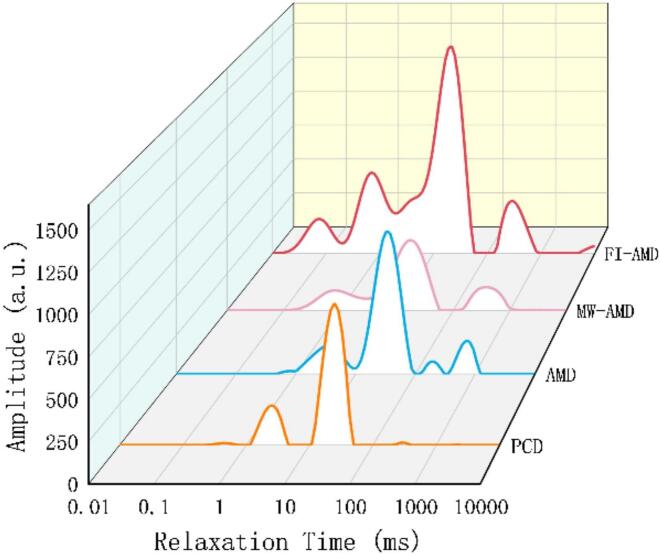
LF-NMR T_2_ relaxation time distribution curves of gluten-free corn dough with different treatments.

LF-NMR T_2_ relaxation measurement was used to analyze water distribution, binding state and mobility in dough systems. Shorter T_2_ relaxation time means stronger water matrix interaction and lower water mobility. T_2_ relaxation time distribution curves are displayed in Fig. 9, and quantitative characteristic parameters are summarized in Table 2. Four water fractions were identified. T_21_ (0.071–3.511 ms) represents tightly bound water bound to internal hydration sites of zein molecules. T_22_ (18.738–32.745 ms) refers to immobilized water trapped in protein networks and starch gels. T_23_ and T_24_ are highly mobile free water filling matrix gaps (Dufour et al., 2024).

**Table 2:** Effect of different zein treatments on LF-NMR T_2_ relaxation characteristic parameters of gluten-free corn dough.

Sample	T_21_/(ms)	T_22_/(ms)	T_23_/(ms)	T_24_/(ms)	A_21_/(%)	A_22_/(%)	A_23_/(%)	A_24_/(%)
PCD	0.43 ± 0.04^c^	24.77 ± 0.98^b^	265.61 ± 12.04^d^	2154.44 ± 52.37^a^	1.36 ± 0.10^a^	98.41 ± 2.76^a^	1.84 ± 0.10^d^	0.29 ± 0.04^d^
AMD	3.51 ± 0.30^a^	32.75 ± 1.44^a^	533.67 ± 22.74^a^	705.48 ± 30.13^c^	1.08 ± 0.08^b^	96.35 ± 2.58^b^	3.43 ± 0.18^a^	0.81 ± 0.06^a^
MW - AMD	0.57 ± 0.05^b^	18.74 ± 0.86^d^	464.16 ± 20.14^b^	ND	1.24 ± 0.09^a^	98.05 ± 2.71^a^	2.83 ± 0.16^b^	0.61 ± 0.05^b^
FI - AMD	0.07 ± 0.01^d^	21.54 ± 0.94^c^	305.39 ± 13.35^c^	1001.00 ± 32.06^b^	1.28 ± 0.09^a^	98.53 ± 2.79^a^	2.25 ± 0.12^c^	0.48 ± 0.04^c^

Note: Different letters within the same column indicate significant differences (*P* < 0.05).

FI-AMD possessed the highest immobilized water proportion (A_22_ = 98.53%), followed by PCD (98.41%), MW-AMD (98.05%) and AMD (96.35%). This result proves that far-infrared-acid synergistic modification best increases immobilized water in corn dough (Dufour et al., 2023). For tightly bound water T_21_, FI-AMD showed the shortest relaxation time (0.071 ms). This value was significantly lower than those of other samples (*P* < 0.05). Far-infrared-acid treatment maximizes zein unfolding. It exposes embedded internal hydration sites and strengthens protein hydration (Uribe-Wandurraga et al., 2019). AMD exhibited the longest relaxation time and the highest total free water proportion (A_23_ + A_24_ = 4.24%). In contrast, FI-AMD had the lowest free water proportion (2.73%). Single acid modification only unfolds zein molecules. It cannot form a dense cross-linked network to fix water molecules. Thermal acid synergistic treatments enhance intermolecular cross-linking. These treatments convert free water into immobilized water (Yang et al., 2019). FI-AMD had slightly lower SS content than MW-AMD (Section 3.2). Nevertheless, its low free water proportion highlights the importance of far-infrared-induced non-covalent interactions. These interactions improve the water-holding capacity of dough (Lefèvre et al., 2024).

### 3.6 Effects of different modifications on thermal stability of dough

DSC was used to characterize the thermal behavior of starch gelatinization and protein cross-linking in dough systems. A higher thermal denaturation peak temperature (T_p_) reflects greater thermal stability of the system. The magnitude of exothermic enthalpy (|−ΔH|) represents the total heat released during thermal transition. This parameter is closely associated with ordered macromolecular structure content and intermolecular interaction strength. A smaller half-peak width (T_c_) suggests higher homogeneity of the macromolecular system and more synchronous thermal transition (Witczak et al., 2012).

**Fig. 10:**
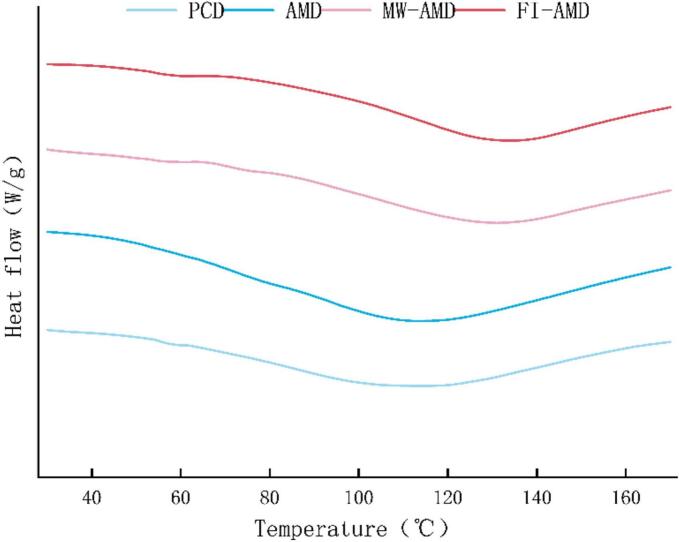
DSC heat flow curves of corn dough incorporated with differently treated zein.

DSC heat flow curves are displayed in Fig. 10. Corresponding thermal parameters are listed in Table 3. The exothermic peak of PCD appeared entirely in the low temperature region, with the broadest peak shape. These features indicate low thermal stability of pure corn dough. By contrast, all zein-modified samples exhibited exothermic peaks shifting toward the high temperature region.

**Table 3:** DSC characteristic temperature and enthalpy parameters of corn dough with different treatments.

Sample	T_s_(°C)	T_0_(°C)	T_p_(°C)	T_c_(°C)	T_e_(°C)	−ΔH (J/g)
PCD	29.64 ± 1.46^a^	52.99 ± 0.60^c^	109.39 ± 0.17^d^	67.58 ± 0.50^b^	115.10 ± 0.30^d^	14.59 ± 0.30^b^
AMD	23.97 ± 1.60^b^	49.21 ± 0.77^d^	111.23 ± 0.06^c^	69.70 ± 0.43^a^	117.00 ± 0.25^c^	20.58 ± 0.40^a^
MW-AMD	15.87 ± 0.89^c^	78.57 ± 0.58^b^	128.75 ± 1.06^b^	54.39 ± 0.04^c^	135.00 ± 0.40^b^	10.84 ± 0.03^c^
FI-AMD	14.46 ± 0.49^c^	89.57 ± 0.07^a^	131.21 ± 0.65^a^	45.47 ± 0.03^d^	138.50 ± 0.15^a^	8.19 ± 0.04^d^

Note: Different letters within the same column indicate significant differences (*P* < 0.05).

The FI-AMD group had the highest T_0_, T_p_ and T_e_ among all samples. The three values increased by 69.0%, 19.9% and 20.3% respectively, compared with the PCD group. This result confirms that far-infrared-acid synergistic modification greatly improves the thermal stability of the dough system. This conclusion matches well with two previous results: the highest storage modulus in the rheological test, and the highest immobilized water proportion in the LF-NMR test (Section 3.5). The FI-AMD group had the smallest T_c_. This indicates the best macromolecular homogeneity and the most synchronous thermal transition (Zhang et al., 2025). Its exothermic enthalpy (−ΔH = 8.19 J/g) was the lowest among all samples. This is due to sufficient cross-linking and structural rearrangement of zein molecules during modification. These changes greatly reduce the exothermic reaction caused by further cross-linking and ordered structure formation in subsequent heating (Tang et al., 2009). In addition, the compact protein network restricts the swelling and gelatinization of starch granules. It further reduces the total heat flow change of the system. This leads to a significantly lower -ΔH. The MW-AMD group showed the same thermal characteristic trend as the FI-AMD. Its thermal stability was significantly better than that of PCD and AMD. But its improvement effect was weaker than that of FI-AMD.

### 3.7 Effects of different modifications on dynamic rheological properties of dough

**Fig. 11:**
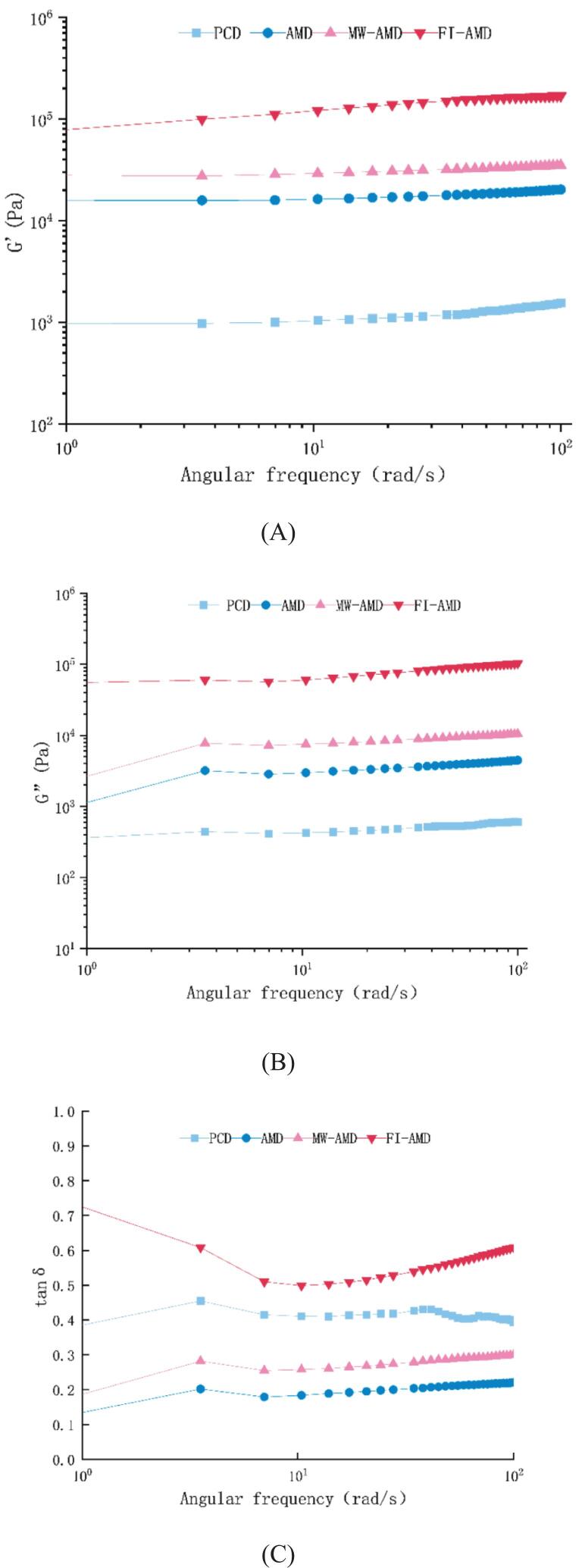
Dynamic viscoelasticity of gluten-free corn dough with different treatments (Fig. 11. (A), Fig. 9(B), and Fig. 9(C) represent the G', G", and tan δ plots, respectively.)

The frequency dependence of G′, G′′ and tanδ of all samples is shown in Fig. 11. Among all samples, FI-AMD achieved the highest G' (ranging from 10^5^ to 2 × 10^5^ Pa) and G′′ (ranging from 5 × 10^4^ to 10^5^ Pa), demonstrating the most robust elastic network and the strongest energy dissipation capacity (Liu et al., 2023). This was followed by MW-AMD (G′: ∼3 × 10^4^–5 × 10^4^ Pa; G′′: ∼10^4^–1.5 × 10^4^ Pa), AMD (G′: ∼1.5 × 10^4^–2 × 10^4^ Pa; G′′: ∼3 × 10^3^–5 × 10^3^ Pa), while PCD presented the lowest moduli (G′: ∼10^3^–2 × 10^3^ Pa; G′′: ∼5 × 10^2^–10^3^ Pa). These results confirm that thermal-acid modification of zein significantly enhances the viscoelasticity of gluten-free dough. Far-infrared-acid treatment has the most significant improvement effect (Timmermans et al., 2022).

All samples maintained an elastic-dominant behavior (tanδ <1) throughout the tested frequency range. The AMD sample had the minimum tanδ at ∼0.2–0.22. This result revealed that AMD contained the highest proportion of elastic components. Single acid treatment facilitated the construction of rigid protein networks inside dough. Nevertheless, insufficient cross-linking density limited the overall modulus of AMD (Iacovino et al., 2024). The tanδ of FI-AMD reached the maximum range of ∼0.6–0.7 among all dough samples. Although its storage and loss moduli were much higher than other groups, this treatment still achieved coordinated elastic and viscous performance. Such rheological characteristics corresponded to its superior balance of tensile strength and ductility observed in tensile measurements (Hackenberg et al., 2018). MW-AMD had a moderate tanδ (∼0.25–0.3) with balanced viscoelastic performance, while PCD maintained a stable tanδ (∼0.4–0.45) across the entire frequency sweep.

### 3.8 Effects of different modifications on textural and tensile properties of dough

Texture profile analysis (TPA) evaluates the processing properties of gluten-free corn dough, including hardness, adhesiveness, springiness, cohesiveness and chewiness (Table 4) (Bchir et al., 2013). Compared with PCD, AMD had higher springiness, cohesiveness, adhesiveness and chewiness. Its hardness dropped from 454.22 g to 272.87 g. Single acid modification unfolded zein molecules and exposed reactive sites. However, it could not trigger enough intermolecular cross-linking. A compact three-dimensional network failed to form accordingly. The dough lost supporting force and became softer (Vilmane & Straumite, 2014). Thermal-acid synergistic modification can comprehensively improve multiple textural indicators of corn dough. The FI-AMD group achieved the highest values of all texture parameters among all tested samples. Its hardness was 866.20 g, adhesiveness was 26.77, springiness was 0.65 and cohesiveness was 0.39. These four indicators increased by 90.7%, 436.5%, 209.5% and 85.7% respectively when compared with PCD. Far-infrared-acid synergistic treatment significantly strengthens dough support capacity, cohesiveness and deformation resistance. This finding shows good consistency with rheological measurement results. FI-AMD displayed the maximum storage modulus (G′) in the previous rheological test (Wang et al., 2017) (Section 3.7).

**Table 4:** Texture profile analysis (TPA) parameters of gluten-free corn dough with different treatments.

Sample	Hardness(g)	Adhesiveness	Springiness	Cohesiveness	Chewiness
PCD	454.22 ± 1.61^c^	4.99 ± 0.07^d^	0.21 ± 0.01^d^	0.21 ± 0.01^d^	25.66 ± 0.34^d^
AMD	272.87 ± 2.53^d^	6.61 ± 0.01^c^	0.40 ± 0.02^c^	0.28 ± 0.01^c^	553.53 ± 0.40^b^
MW-AMD	618.80 ± 0.36^b^	13.71 ± 0.01^b^	0.47 ± 0.01^b^	0.36 ± 0.01^b^	765.67 ± 0.66^a^
FI-AMD	866.20 ± 5.18^a^	26.77 ± 0.27^a^	0.65 ± 0.01^a^	0.39 ± 0.01^a^	219.37 ± 4.51^c^

Note: Different letters within the same column indicate significant differences (*P* < 0.05).

**Fig. 12:**
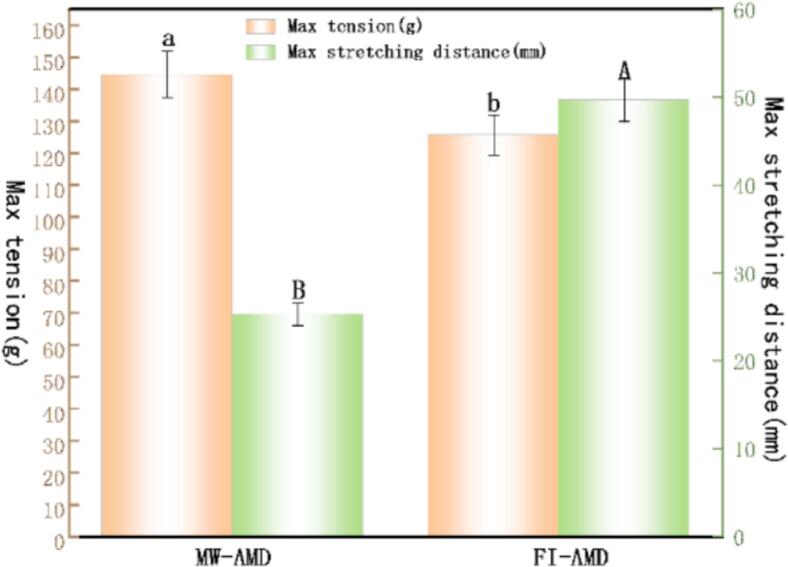
Maximum tensile resistance and maximum stretching distance of gluten-free corn dough samples.

The tensile properties of dough are shown in Fig. 12. The MW-AMD group had a significantly higher maximum tensile resistance (145.3 ± 4.3 g) than the FI-AMD group (127.5 ± 3.8 g, *P* < 0.05). MW-AMD contained higher density of intermolecular disulfide cross-linking. This structure provided dough with stronger rigidity and anti-deformation capacity. The tensile strength of the whole dough matrix was therefore enhanced (McCann et al., 2016). By contrast, FI-AMD possessed a much larger maximum stretching distance of 49.7 ± 1.5 mm. MW-AMD only reached 25.1 ± 1.1 mm, and the difference was statistically significant (*P* < 0.05). The stretching distance of FI-AMD was almost twice that of MW-AMD. Far-infrared-acid modification effectively improved the ductility and flexibility of corn dough. No valid tensile test data were obtained from PCD and AMD samples. Two key factors led to this result. For the PCD group, starch granules were relatively large. The interfacial bonding between starch and zein was weak. In addition, high amylose content made the dough naturally brittle. Thus, PCD failed to form an extensible continuous network. For the AMD group, acid treatment only partially unfolded zein molecules, and could not generate enough intermolecular cross-linking (including disulfide bonds and non-covalent interactions) to build a continuous viscoelastic protein network. MW-AMD and FI-AMD exhibited distinct functional performances. MW-AMD showed greater tensile strength. This characteristic makes it suitable for baking applications that demand high dough toughness. By comparison, FI-AMD achieved a favorable combination of moderate tensile strength and superior ductility.

### 3.9 Correlation analysis between protein structure and dough quality

**Fig. 13:**
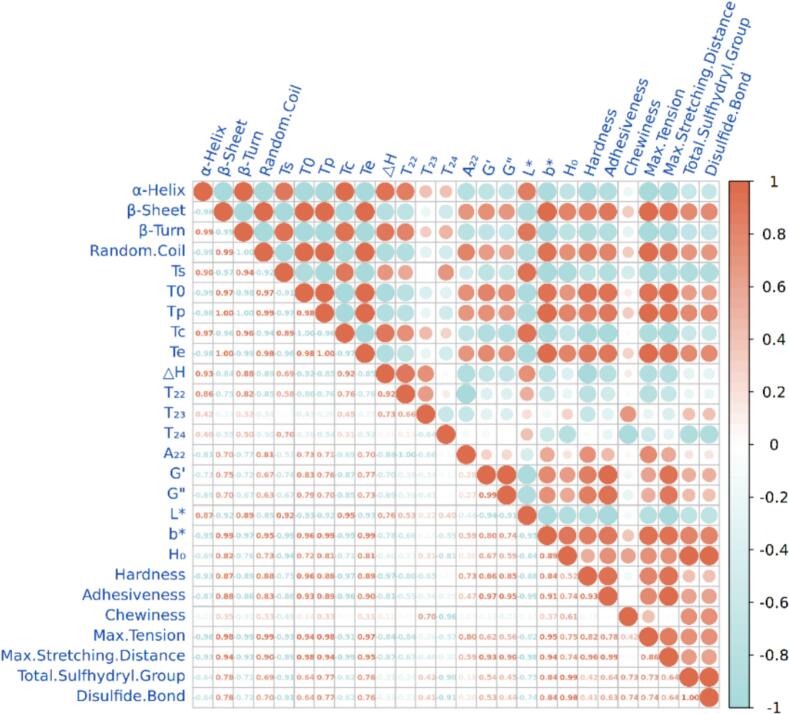
Pearson correlation heatmap of core indicators in gluten-free dough. Note: The color intensity and circle size represent the magnitude of the correlation coefficient (r), with red indicating positive correlation and blue indicating negative correlation. The numbers inside the circles are the correlation coefficients.

The Pearson correlation heatmap of all indicators is shown in Fig. 13. An extremely significant negative correlation was found between α-helix and β-sheet content (r ≈ −1, *P* < 0.01), while an extremely significant positive correlation existed between α-helix and β-turn content (r ≈ 0.99, *P* < 0.01). These results confirmed that zein modification disrupted the compact and ordered α-helix and β-turn conformations of native protein and induced their ordered conversion into β-sheet structures. β-Seet content showed extremely significant positive correlation with dough thermal stability (T_p_), rheological properties (G^'^), textural properties (hardness) and stretching ductility (*r* > 0.9, *P* < 0.01), while α-helix content was extremely significantly negatively correlated with all these processing indicators (*P* < 0.01). This demonstrated that the formation of ordered β-sheet structure is the basis for the quality improvement of gluten-free dough. The immobilized water proportion A_22_ was significantly positively correlated with dough hardness and β-sheet content (*P* < 0.05), and extremely significantly negatively correlated with the relaxation time T_22_ (r ≈ −0.995, *P* < 0.01), confirming that the compact protein network can effectively immobilize water and optimize the water holding capacity of dough. Surface hydrophobicity showed an extremely significant positive correlation with total sulfhydryl and disulfide bond content (r ≈ 0.99, *P* < 0.01), which verified the molecular pathway of thermal-acid modification: promoting zein molecular unfolding, exposing internal hydrophobic groups and free sulfhydryl groups, and enhancing protein network cross-linking via intermolecular disulfide bond formation.

### 3.10 Regulatory mechanism of thermal-acid modifications on gluten-free dough quality

**Fig. 14:**
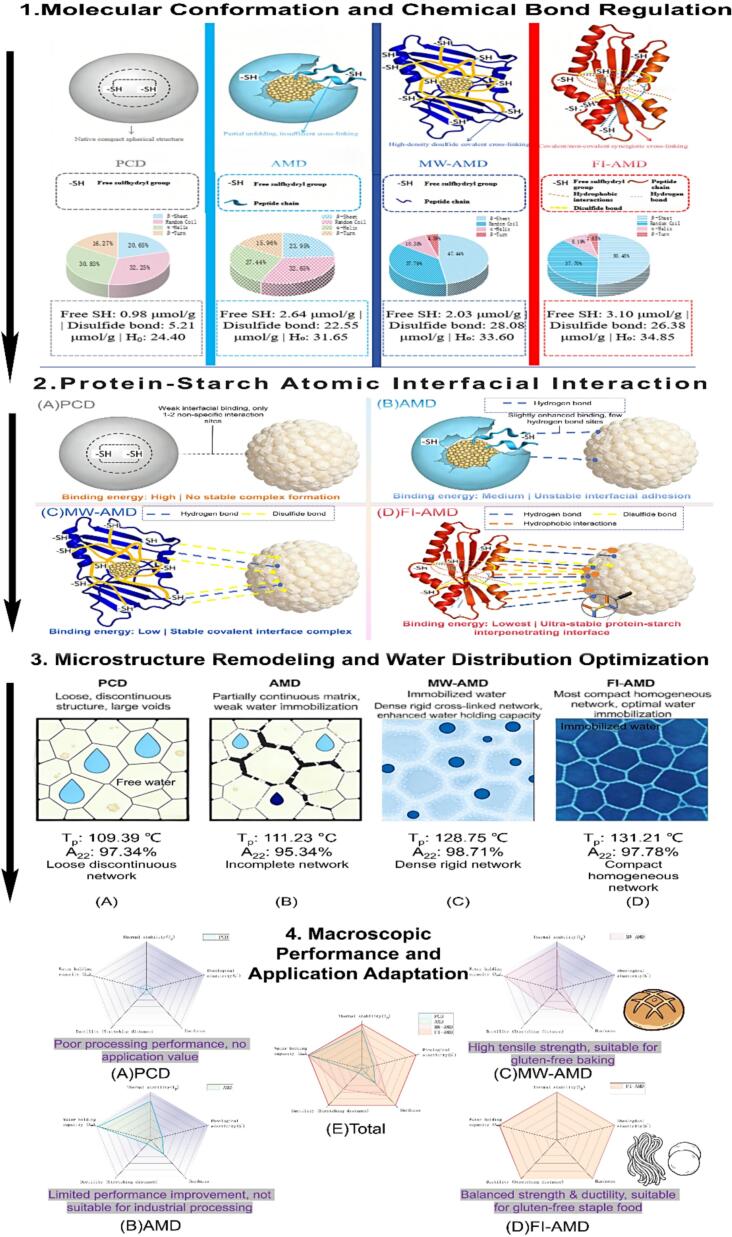
Schematic illustration of the regulation mechanism of microwave-acid and far-infrared-acid synergistic modification on gluten-free zein-corn dough.

Based on the above results, the differential regulatory mechanism of microwave-acid and far-infrared-acid synergistic modification on gluten-free corn dough was elucidated, as shown in the schematic diagram (Fig. 14). Correlation analysis confirmed that β-sheet content was extremely significantly positi*v*ely correlated with dough thermal stability, rheological modulus, hardness and stretching ductility (r > 0.9, *P* < 0.01), which indicated that the conformational transformation of zein from α-helix to β-sheet is the molecular basis for dough quality improvement. In the microwave-acid treatment, rapid volumetric heating creates instantaneous high temperatures within protein particles. This triggers fast unfolding of zein molecules, exposes abundant free sulfhydryl groups embedded in the hydrophobic core, and accelerates sulfhydryl oxidation to form dense intermolecular disulfide covalent cross-links. The dense covalent cross-linking network constructs a rigid and continuous protein matrix wrapping starch granules, which significantly enhances the tensile strength and toughness of the dough. For far-infrared-acid treatment, the uniform and mild radiative heating effect can resonate with the functional groups of zein molecules, which maximizes the unfolding of protein chains without causing excessive instantaneous aggregation, and simultaneously exposes free sulfhydryl groups and hydrophobic amino acid residues, thus achieving the synergistic enhancement of covalent disulfide cross-linking and non-covalent interactions (hydrogen bonds, hydrophobic interactions). This synergy further accelerates the α-helix-to-β-sheet transition; in the present study, the β-sheet fraction reached a maximum value of 50.46%. The ordered β-sheet structure forms a compact protein network to guarantee dough strength. Meanwhile, reversible modulation of non-covalent interactions imparts outstanding ductility and flexibility, realizing an optimal balance between strength and extensibility. The two thermal treatments modulate zein conformation through distinct mechanisms, constituting the fundamental basis for their divergent effects on dough quality. Moreover, this study elucidates a comprehensive regulatory cascade: (i) conformational rearrangement of zein proteins; (ii) strengthening of intermolecular protein–protein interactions; (iii) enhanced interfacial adhesion between zein and starch; (iv) reconstruction of the dough microstructural architecture; and (v) consequent optimization of macroscopic processing properties.

## 4 Conclusion

This study revealed the differential regulatory effects of microwave-acid and far-infrared-acid synergistic modification on zein structure and gluten-free corn dough quality. Both thermal-acid treatments significantly optimized the conformational properties of zein and the processing performance of dough. Microwave-acid treatment mainly enhanced dough tensile strength via high-density disulfide cross-linking, while far-infrared-acid treatment achieved an excellent balance between dough strength and ductility through covalent and non-covalent synergistic interactions. This work provides a theoretical basis for the development of high-quality gluten-free cereal staple foods.

## CRediT authorship contribution statement

**Yanjia Liu:** Writing – original draft, Visualization, Investigation, Formal analysis, Data curation, Conceptualization. **Zhuang Liu:** Writing – review & editing, Validation, Investigation, Formal analysis. **Bin Song:** Methodology. **Qianqian Li:** Investigation. **Yu Wang:** Software. **Yuhan Zhou:** Investigation. **Chengbin Zhao:** Project administration. **Hao Zhang:** Project administration. **Yuzhu Wu:** Writing – review & editing, Visualization, Supervision. **Xiuying Xu:** Project administration, Funding acquisition. **Jingsheng Liu:** Validation, Funding acquisition.

## Declaration of competing interest

The authors declare that they have no known competing financial interests or personal relationships that could have appeared to influence the work reported in this paper.

## Data Availability

Data will be made available on request.
